# The 3D Language of Cancer: Communication via Extracellular Vesicles from Tumor Spheroids and Organoids

**DOI:** 10.3390/ijms26157104

**Published:** 2025-07-23

**Authors:** Simona Campora, Alessandra Lo Cicero

**Affiliations:** Department of Biological, Chemical and Pharmaceutical Sciences and Technologies (STEBICEF), University of Palermo, 90128 Palermo, Italy

**Keywords:** extracellular vesicles, exosomes, spheroids, organoids, tumor

## Abstract

Extracellular vesicles (EVs) have emerged as key mediators of intercellular communication, gaining recognition as tumor biomarkers and promising therapeutic targets. As the study of EVs advances, it has become increasingly clear that the cellular context in which they are produced significantly influences their composition and function. Traditional two-dimensional in vitro models are being progressively replaced by more advanced three-dimensional systems, such as tumor spheroids and organoids. These 3D models are particularly valuable in cancer research, providing a more accurate representation of the complex cellular and molecular heterogeneity that characterizes tumors, better mimicking the in vivo microenvironment compared to standard monolayer cultures. This review explores the role of EVs derived from tumor spheroids and organoids in key oncogenic processes, including tumor growth, metastasis, and interactions within the tumor microenvironment. We highlight how EVs contribute to the spread of cancer cells, affecting surrounding tissues, and promote immune evasion, which poses significant challenges in cancer therapy.

## 1. Introduction

Extracellular vesicles (EVs) are ubiquitous, membrane-bound nanosized particles involved in various physiological and pathological processes [1,2]. They play crucial roles in intercellular communication by delivering bioactive cargos, such as proteins, lipids, and nucleic acids, that can significantly influence target recipient cells, organs, and the surrounding microenvironment [3,4]. Due to these properties, EVs are being explored as promising biomarkers, therapeutic agents, and Drug Delivery Systems (DDSs).

Although EVs play essential homeostatic functions, they are also implicated in different diseases, including cancer, which is a major cause of worldwide mortality. According to the World Health Organization, nearly 20 million new cancer diagnoses and 10 million cancer-related deaths were reported in 2023, with cases continuing to increase annually. Many studies have reported that cancer cells secrete higher quantities of EVs compared to non-malignant cells. These vesicles are actively involved in promoting tumor progression by facilitating invasion, metastasis, angiogenesis, and immune evasion [5,6].

Although monolayer cell cultures are widely employed for isolating EVs due to their affordability and workability, they fail to accurately replicate the structural and functional complexity, resulting in EVs that lack authentic pathological characteristics observed in vivo [7,8]. Tumor masses consist of cancer cells and immune and stromal cells, producing a typical dense extracellular matrix (ECM), which influences EV production and composition [9]. Therefore, a more representative model for isolating EVs was investigated by developing different 3D cell culture models, which often also include ECM components. For this purpose, both adherent and non-adherent approaches have been explored for growing cells in 3D structures [10]. Adherent methods included the use of scaffolds (natural or synthetic polymers, hydrogels, decellularized tissues), microfluidics, and bioreactors [11]. While these models offer advantages, including long-term culture, homogeneous aggregates, and controlled cell number and viability, they also present some disadvantages, including difficulties in isolating cells and EVs and removing residual serum, resulting in the contamination of serum-derived vesicles, which complicates the purification of cell-specific EVs. These limitations can be overcome by non-adherent techniques, such as hanging drop and liquid overlay techniques, which form tumor spheroids and organoids. Spheroids effectively mimic key tumor characteristics, including cell–cell and cell–matrix interactions, the nutrients and oxygen gradients, and the heterogeneity typical of solid tumors. On the other hand, organoids are more complex systems that include additional vascular components and are enriched with more representative extracellular compounds. The choice of the appropriate 3D system depends on the specific aim and application.

This review provides a comprehensive analysis of the molecular composition and functional roles of EVs released by tumor spheroids and organoids. It highlights their involvement in tumor growth, their role in modulating the tumor microenvironment, and their potential as biomarkers and therapeutic targets in cancer treatment.

## 2. Biogenesis and Characteristics of Extracellular Vesicles

**Figure 1:**
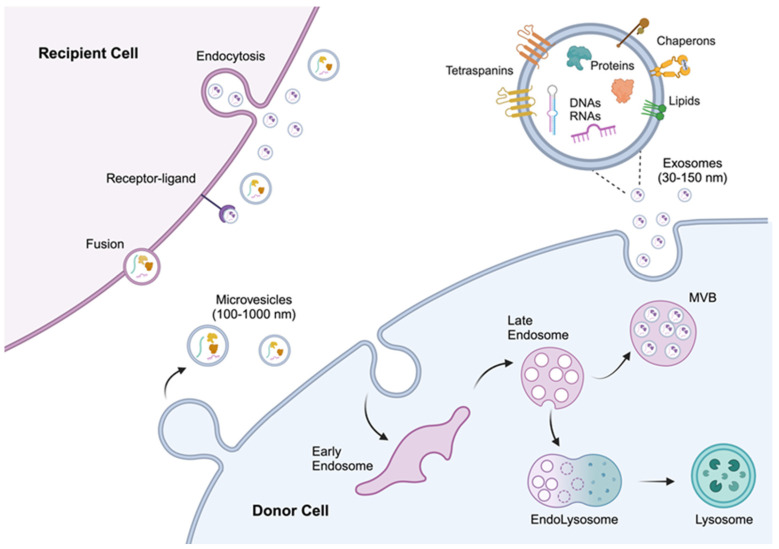
Extracellular vesicle biogenesis. Two main types of EVs are distinguished based on their biogenesis. Exosomes (30–150 nm) are generated through the endocytic pathway and are released via exocytosis of the multivesicular bodies (MVBs). Late endosomes either fuse with lysosomes for degradation or fuse with the plasma membrane to release the intraluminal vesicles that are called exosomes. Microvesicles (100–1000 nm) are released through budding from the plasma membrane. EVs can be internalized by recipient cells through endocytosis, receptor-mediated signaling pathways, or membrane fusion.

EVs are mainly generated through two mechanisms: the blebbing of the plasma membrane (microvesicles) or within multivesicular bodies (MVBs), followed by release by exocytosis as exosomes [12,13]. Multiple mechanisms contribute to vesicle formation, influencing their heterogeneity, size, and function. Exosomes and microvesicles consist of a mixture of membrane and cytosolic elements, including proteins, lipids, and RNAs, and their composition varies based on their biogenesis site [1,14] (Figure 1).

Exosomes are the smallest type of EVs, typically ranging from 30 to 150 nanometers in diameter. They originate from the endosomal system through the inward budding of the plasma membrane, leading to the formation of early endosomes, which mature into late endosomes. These can follow two different pathways: they may fuse with lysosomes, resulting in the degradation of their contents, or they may give rise to MVBs [15]. During MVB maturation, the endosomal membrane undergoes inward budding, forming mature intraluminal vesicles (ILVs). Different molecular cargoes are selectively sorted into ILVs in the late endosomes through specific signaling pathways and molecular machinery. In this context, the Endosomal Sorting Complex Required for Transport (ESCRT) plays a crucial role in ILV biogenesis [16,17], comprising several protein subcomplexes (ESCRT-0, ESCRT-I, ESCRT-II, and ESCRT-III) that sequentially assemble on the endosomal membrane to facilitate cargo trafficking and vesicle budding [16,17,18]. Additionally, sorting molecules such as tetraspanins and specific lipids like ceramides assist in cargo selection [19], while transmembrane proteins, including CD9, CD63, and CD81, contribute to the organization of microdomains on the endosomal membrane [20,21,22]. MVBs ultimately fuse with the plasma membrane to release the ILVs as exosomes into the extracellular space. This process is regulated by several factors, including Rab GTPases (e.g., Rab27a and Rab27b), soluble NSF (N-ethylmaleimide-Sensitive Factor) attachment receptor (SNARE) proteins, and other regulatory factors [23,24].

Microvesicles (MVs) are typically larger than exosomes, ranging from 100 to 1000 nanometers in diameter. They are formed through the direct outward budding and fission of the plasma membrane, a process that involves the rearrangement of lipid and protein components [13,14,15]. This remodeling is often driven by phospholipid redistribution, such as the externalization of phosphatidylserine, which plays a pivotal role in membrane curvature and budding during both exosome and microvesicle formation [19,25]. Additionally, the reorganization of the actin cytoskeleton, regulated by small GTPases like ARF6 and RhoA, is essential in this process. Specifically, these small molecules modulate the contractile machinery necessary for MV fission and release into the extracellular environment [26].

Both exosomes and MVs are complex structures carrying a wide variety of biomolecules, reflecting the status and function of their originating cells. The membranes of EVs have a specific lipidic composition that is rich in phospholipids like phosphatidylserine, phosphatidylcholine, and phosphatidylethanolamine, and is involved in membrane curvature and budding. Moreover, sphingomyelin and ceramides contribute to membrane structure and dynamics [27,28], while cholesterol is involved in maintaining the stability and fluidity of the EV membrane [29]. Furthermore, the EV membrane contains tetraspanins (e.g., CD9, CD63, CD81), integrins, and various receptors crucial for their formation, release, targeting, and uptake by recipient cells.

On the other hand, the inner molecular composition typically includes proteins, lipids, nucleic acids, heat shock proteins (HSP70, HSP90), cytoskeletal proteins (actin, tubulin), and enzymes (GAPDH, enolase), contributing to the vesicle function and intercellular communication abilities. For instance, EVs can carry proteins involved in signaling pathways, including kinases, transcription factors, and growth factors, which modulate cellular responses in recipient cells [20,21,22,30]. Additionally, EVs can transport different classes of RNA, including messenger RNAs (mRNA) translated into proteins in recipient cells, potentially influencing their gene expression profile [31,32]; microRNAs, like small and non-coding RNAs, which regulate gene expression post-transcriptionally and are involved in processes such as cell proliferation, differentiation, and apoptosis [33,34,35,36,37]; and long non-coding RNAs (lncRNAs) and small nucleolar RNAs (snoRNAs), which may also modulate gene expression and cellular functions [38,39]. EVs can also contain fragments of double-stranded DNA (dsDNA) reflecting the genetic material of the parent cell [40,41]. Furthermore, mitochondrial DNA (mDNA) can be packaged and transferred into exosomes, potentially impacting the metabolic and signaling pathways of recipient cells [42,43]. Moreover, mitochondria can also release exosome-like vesicles themselves [44].

Although the process of EV biogenesis is similar in healthy and tumor cells [45], the molecular composition can significantly depend on the donor cell type. For instance, tumor-derived EVs can transfer oncogenic factors like mutated proteins or miRNA that contribute to tumor growth [46,47], invasion [48], formation of pre-metastatic niches [49], and metastasis [50]. Furthermore, these EVs can also carry molecules associated with drug resistance [51,52] and immuno evasion [53], promoting a lack of therapeutic efficacy and tumor progression.

## 3. Isolation and Characterization Techniques

To study and exploit the potential role of different EVs, several steps, including isolation, purification, and characterization, are required [54,55]. Accurate and reliable techniques are crucial for separating distinct EV populations and ensuring the purity and integrity of the vesicles for downstream applications, such as proteomic, genomic, and functional analyses [56]. Various methods have been employed for this purpose, each with its advantages and limitations [57,58,59]. Differential ultracentrifugation, often regarded as the gold standard for the isolation of EVs [60,61], involves sequential centrifugation steps at increasing speeds to remove cells, debris, and large vesicles, followed by ultracentrifugation at high speeds to pellet EVs. Density gradient purification, which utilizes a continuous density gradient of sucrose or iodixanol in combination with differential centrifugation, achieves higher purity levels for isolating EVs, which may be combined with other methods, such as nanomembrane-based filtration [62]. Since EVs contain protein makers indicative of their cellular origin, specific EV subtypes can be collected by targeting peculiar surface proteins using immunoaffinity-based approaches [62,63]. For instance, common biomarkers for EV isolation include tetraspanins like CD9, CD63, and CD81.

After isolation and enrichment, EVs can be characterized in terms of size and concentration using techniques like Nanoparticle Tracking Analysis (NTA) [64] and Transmission Electron Microscopy (TEM) [65,66]. Their molecular cargo can be further investigated through methods like flow cytometry [67,68], mass spectrometry [69,70], and RNA-seq datasets for miRNAs, lncRNAs, and mRNAs [71].

## 4. Tumor-Derived EVs

**Figure 2:**
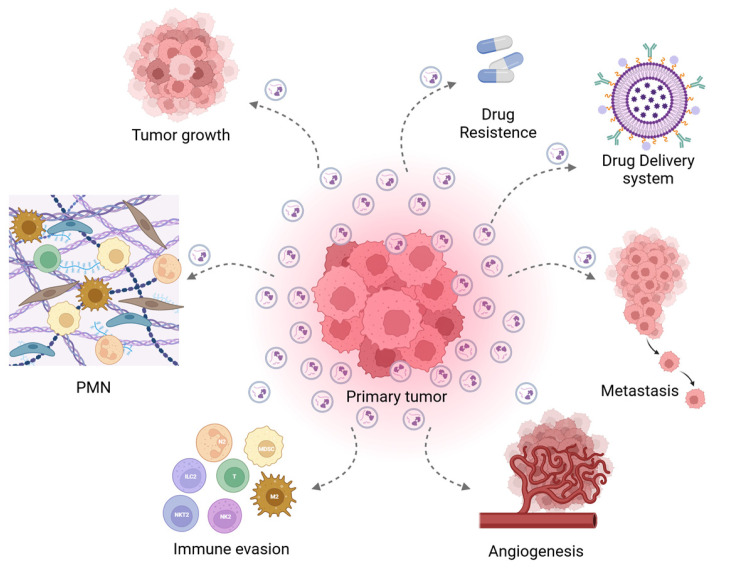
Comprehensive role of EVs in cancer. Tumor-derived EVs contribute to multiple cancer-related processes, including tumor growth, pre-metastatic niche (PMN) formation, immune evasion, angiogenesis, metastasis, drug resistance, and drug delivery.

EVs play a pivotal role in intercellular communication under both physiological and pathological conditions [72]. Cancer cells produce and release higher quantities of EVs compared to normal cells, often containing altered cargo specifically adapted to support tumor heterogeneity and adaptability. The impact of EVs is highly context-dependent, varying with tumor type, stage, and microenvironmental conditions. In many cancers, EVs facilitate proliferation, invasion, angiogenesis, and metastasis. These vesicles disseminate molecular signals both locally and systemically, contributing to the maintenance and progression of the primary tumor mass [73,74]. Numerous studies have demonstrated EVs’ ability to carry bioactive molecules, promoting cancer development and progression [37,75,76]. Within the tumor microenvironment, EVs released by various cells —such as cancer cells, immune cells, and stromal cells—contribute to oncogenic processes, including tumor growth, angiogenesis, immune evasion, and metastasis [33,77,78,79,80,81,82] (Figure 2).

EVs contribute to cancer progression by promoting the formation of pre-metastatic niches (PMNs), a specialized microenvironment that prepares for the implantation of organ-specific metastasis [83]. EVs can alter the vasculature and accumulation of bone marrow-derived cells (BMDCs) to enhance metastatic colonization, as observed in melanoma [84]. Tumor-derived EVs have been shown to establish immunosuppressive PMNs by impairing Natural Killer (NK) cell activity and inhibiting the maturation of dendritic cells (DC). García-Silva and colleagues demonstrated that melanoma-secreted EVs promote lymphangiogenesis and lymph node metastasis through the nerve growth factor (NGF) receptor (NGFR), which acts on lymphatic endothelial cells [85]. A tumor mass is a complex of different types of cells, including cancer cells, carcinoma-associated fibroblasts (CAFs), adipocytes, stem cells, endothelial cells, and immune cells [86]. EVs modulate tumor vasculature by delivering pro-angiogenic factors, such as vascular endothelial growth factor (VEGF) [87], and upregulating the expression of the vascular endothelial growth factor receptor FLT1 [81] in endothelial cells, which stimulates angiogenesis, enhances vascular permeability, and facilitates the formation of pre-metastatic niches. These vascular changes enable key steps in the metastatic process, including tumor cell intravasation, extravasation, and metastatic colonization. Moreover, CAF-derived EVs in particular play a critical role in PMN formation, as suggested by Kong et al. They demonstrated that CAF EV uptake by lung fibroblasts is mediated by integrin α2β1, a fundamental process for cell activation, PMN development, and metastasis formation [87]. However, multiple other integrin subtypes are also implicated in EV-driven tumor progression. EVs exhibit tissue-specific accumulation patterns that correlate with future metastatic sites; notably, EVs expressing α6β4 and α6β1 integrins are linked to lung metastasis, while those bearing αvβ5 integrin preferentially target the liver [81]. Moreover, EVs released by cancer stem cells differ in molecular composition from those released from differentiated cancer cells. This has been exemplified in the triple-negative breast cancer (TNBC) model in which EVs secreted by cancer stem cells drive PMN remodeling in both in vitro and in vivo systems [88].

Additionally, EVs can modulate the immune system by suppressing immune cell activity, thereby creating an immunosuppressive tumor microenvironment [89,90]. EVs also contribute to remodeling the extracellular matrix by altering the surrounding stromal cells’ phenotypes, making them more supportive of cancer cell proliferation and invasion. For instance, cancer cell-derived EVs can reprogram fibroblasts into CAFs, further promoting tumor progression [91,92,93]. A notable example of this mechanism is provided by Yang and colleagues, who demonstrated the crucial role of colorectal cancer (CRC)-derived EVs in the early formation of liver metastasis. Their study revealed that transforming growth factor-beta 1 (TGF-β1) carried by CRC-derived EVs promotes the differentiation of hepatic stellate cells (HSCs) into CAFs. These CAFs recruit myeloid-derived suppressor cells (MDSCs), which inhibit NK activity and establish immunosuppressive PMNs [94].

Furthermore, EVs are implicated in drug resistance, making cells more resilient to conventional therapies [95]. In response to classic chemotherapy, cancer cells, like melanoma cells, secrete more EVs able to restart cancer growth in in vivo systems, specifically by enhancing arginase 1 and IL10 in stromal cells and stimulating the transcription of genes involved in the DNA repair process [96]. EVs also contribute to the drug efflux mechanism, as reported by Yang et al., who demonstrated that EVs promote the expulsion of the temozolomide (TMZ) in glioblastoma (GBM) cells, thereby reducing its cytotoxic efficacy and inducing drug resistance [97]. Programmed cell death protein-ligand 1 (PD-L1) found on the surface of tumor EVs inhibits T cell activation, leading to immune escape mechanisms mediating tumor progression [90,98,99].

Even EVs released by other cancer-associated cells, including CAF or immune cells, play a pivotal role in this process [100,101]. In thyroid cancer, programmed cell death protein 1 (PD-1) therapy resistance is mediated by tumor-associated macrophage (TAM)- derived EVs through the delivery of miR-21-5p, which inhibits methyltransferase-like 3 (METTL3) [102].

On the other hand, EVs represent potential targets for innovative therapeutic strategies due to their crucial role in the intricate network of interactions that drive cancer’s development and progression. Furthermore, they can be functionalized as a drug delivery system by incorporating drugs and antibody-binding moieties specific to the fragment crystallizable (Fc) domain for active tumor targeting [103,104]. For instance, biomarkers, which are measurable indicators of physiological and pathological biological processes, are involved in tracking disease progression and response to treatment. EVs from cancer cells hold significant promise for diagnostics, as they carry specific molecules—such as mutated DNA, proteins, and microRNAs—that serve as biomarkers, reflecting the state and function of their originating cells and, therefore, indicating the presence and progression of cancer, including metastasis [32,105,106]. For instance, the proteins PF4 and AACT in serum EV samples and the two transmembrane proteins CD147 and A33 in fecal EVs are key markers for colorectal cancer [107,108]. By offering an efficient tool for tumor detection, EVs present several advantages, including cost-effectiveness, pain reduction, and the potential to serve as a non-invasive alternative to traditional surgical methods.

## 5. Tumor Spheroids and Organoids: An In Vitro Model

Tumor spheroids and organoids are three-dimensional (3D) cell culture models that provide more physiologically relevant insights into tumor biology than traditional two-dimensional (2D) monolayer cultures [109]. Spheroids are typically derived from single-cell suspensions with self-assembly capabilities to mimic the architecture and microenvironment of solid tumors. This includes replicating complex cellular interactions, cell–matrix interactions, and nutrient and oxygen gradients present in vivo [110,111]. On the other hand, organoids are more complex 3D structures that closely mimic native tumor architecture and function, typically comprising multiple cell types, including elements of the tumor microenvironment (TME) [112].

**Figure 3:**
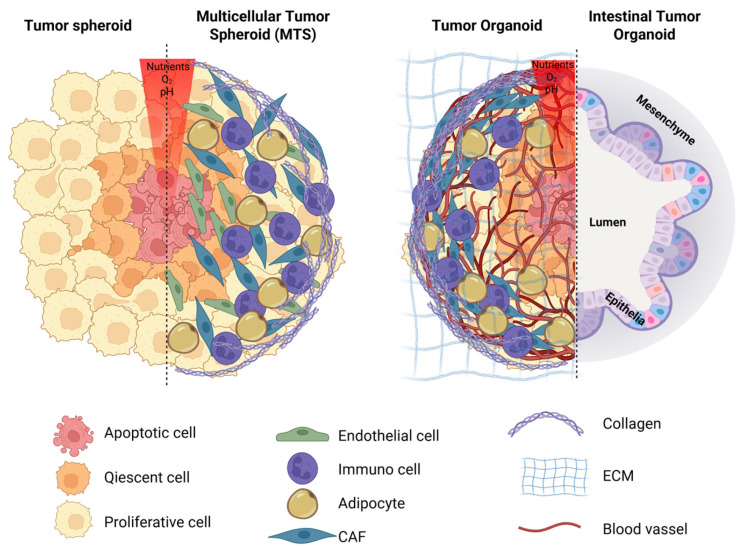
Representative models of tumor spheroids, multicellular tumor spheroids (MCTS), tumor organoids, and intestinal tumor organoids. Tumor spheroids are three-dimensional aggregates of cancer cells, while multicellular tumor spheroids incorporate additional cell types, such as cancer-associated fibroblasts (CAFs), endothelial cells, immune cells, or adipocytes, to better replicate the tumor microenvironment and intercellular interactions. Tumor organoids are more complex structures with well-defined cell organization, vascularization, and an endogenous or additional complex extracellular matrix (ECM). Both models exhibit a characteristic oxygen, pH, and nutrient gradient from the outer proliferative layer to the inner core. The central regions often become hypoxic, closely resembling conditions within solid tumors. Some tumor organoids, like intestinal ones, present a specific polarization or cells with an inner lumen and mesenchymal cells in the external part and do not present the different layers.

Spheroids exhibit an outer proliferative cell layer, an intermediate quiescent zone, and a hypoxic core caused by restricted nutrient and oxygen diffusion (Figure 3) [113,114,115]. This layered formation creates microenvironments within the spheroid, resulting in oxygen, nutrients, and metabolite gradients affecting cellular behavior and drug response. In particular, the hypoxic core provides a platform to explore how reduced oxygen levels influence tumor progression and resistance to therapy [116,117]. These properties make spheroids an invaluable tool for investigating cancer features, progression, drug responses, and therapeutic resistance. The same architecture is also evident in some organoids, depending on the type (generally cancerous ones), size (big organoids), and culture conditions [118].

Spheroids and organoids can be synthesized using liquid-based techniques that prevent cell adhesion to the substrate while promoting cell–cell interactions. The size of the spheroids depends on the initial number of cells. Several methods can be used to culture cells in 3D. Static cultures in ultra-low-attachment plates or flasks are coated with materials such as poly (2-hydroxyethyl methacrylate) (pHEMA), hyaluronic acid (HA), poly-d-lysine, laminin, or agarose to prevent substrate adhesion [119,120]. The Hanging Drop method, on the other hand, exploits gravity to promote cell aggregation within a drop of culture medium suspended from a surface. In dynamic suspension cultures, the constant movement of the cell suspension within the culture medium prevents substrate adhesion and favors cell–cell contacts. In this context, spinner flasks—devices that maintain the suspension through mechanical stirring—and the RCCS/RWV system, a rotating wall vessel bioreactor, are commonly used [121]. In magnetic levitation, cells are incubated with magnetic iron oxide nanoparticles (MIO NPs), which are taken up through endocytosis. The subsequent application of a magnetic field induces cellular aggregation in plates, leading to spheroid formation [122]. Alternatively, spheroids can also be generated directly in the presence of porous microspheres that act as scaffolds and are simply added to the culture medium [123].

Unlike monolayer cells, which grow on flat surfaces and fail to replicate the complex interactions and cellular diversity found in tumors, spheroids and organoids maintain proper cell–cell and cell–matrix interactions. Even the proteomic profile varies when comparing 2D and 3D systems of the same cells. For instance, spheroids of primary breast cancer showed an increase in collagen and matrix metalloproteinase (MMP) expression compared with monolayer cultures, suggesting a more articulated and organized extracellular environment [124]. On the other hand, epigenetic patterns, including methylation and microRNA expression, change significantly between the two systems [125]. This distinction makes them more suitable for accurate tumor behavior modeling.

Furthermore, solid tumors are a complex ecosystem composed not only of cancer cells but also of non-cancerous cells that significantly contribute to tumor microenvironment (TME) formation and tumor progression. These include immune cells, cancer-associated fibroblasts (CAFs), endothelial cells (ECs), pericytes, adipocytes, and neurons (Figure 3). Although the TME cell composition depends on the tumor stage, the type of cancer, and the patient origin, it is well established that these cells play a role in tumor pathogenesis and their crosstalk with cancer cells through secreted molecules carried by vesicles [126]. In this scenario, multicellular tumor spheroids (MTSs), also called assembloids, provide a more realistic model by incorporating different cell types into a 3D structure [127]. These co-culture systems enable a more comprehensive understanding of tumor–immune interactions and the impact of the tumor microenvironment on cancer progression. For instance, pancreatic ductal adenocarcinoma (PDAC) organoids have been used as a model for studying the cytokine-mediated signaling pathways involved in CAF cell activity [128]. Similarly, MTSs of the osteosarcoma cell line and mesenchymal stromal cells (MSCs) have provided insights into cytokine-driven ECM deposition in osteosarcoma [129]. Moreover, more complex spheroids of pancreatic cancer cells, fibroblasts, and endothelial cells are an optimal system to investigate mechanisms of in vivo-like drug resistance [130].

Organoids are 3D miniature structures derived from various stem or tissue sources: embryonic stem cells (ESCs), induced pluripotent stem cells (iPSCs), adult tissue-resident stem cells (ASCs), and cancer-derived cells [131]. Based on their cellular origin, organoids can be broadly classified into two categories: tissue-derived organoids, which originate from direct isolation of stem cells from primary tissues, and organoids generated from somatic or pluripotent stem cells through directed differentiation protocols.

Organoids derived from ESCs or iPSCs are particularly valuable for modeling early human development, including organogenesis and congenital disorders. Those derived from adult stem cells retain many characteristics of their tissue of origin, making them suitable for studying organ-specific physiology, regeneration, and disease pathology [132].

Cancer-derived organoids, especially those established from patient samples, enable researchers to model tumor heterogeneity, genetic mutations, and drug responses in a personalized context. A major focus in current organoid research lies in patient-derived organoids (PDOs), particularly those established from tumor biopsies [133]. PDOs closely preserve the histological architecture, molecular features, and functional behaviors of the original tumors, providing a powerful system for studying tumor biology, genetic heterogeneity, and therapeutic response in a patient-specific context.

Despite their promise, the inherent self-organizing nature of organoids presents challenges in achieving uniformity and reproducibility. As a result, ongoing research is focused on refining culture conditions to produce consistent and stable organoid models for clinical and translational applications [134].

Most tumor organoid cultures currently use Matrigel as an ECM scaffold [135]. However, synthetic matrix alternatives, such as synthetic hydrogels and gelatin methacrylate (GelMA), are increasingly being explored, offering greater control over chemical composition and mechanical properties to enhance the reproducibility and scalability of organoid culture [136,137]. One of the main challenges in developing tumor organoids is to selectively promote tumor cell proliferation while inhibiting the growth of non-tumor cells present in heterogeneous cell suspensions. To address this, careful optimization of the culture medium is essential. This typically involves adjusting the composition of cytokines and growth factors to create a selective environment that favors the survival and expansion of tumor cells [138,139].

A major limitation of 3D spheroid cultures is the development of diffusion gradients as spheroid size increases. Once spheroids exceed 500 μm in diameter, their multilayered architecture restricts the transport of oxygen and nutrients while impairing the clearance of metabolic waste [140]. This frequently leads to central necrosis and compromises overall structural integrity and cellular viability. Despite these well-known physiological constraints, critical experimental parameters—such as oxygen levels, fetal bovine serum (FBS) concentration, media composition (e.g., glucose and calcium content), and initial cell density—are often inconsistently reported or neglected, contributing to poor reproducibility and hindering clinical translation. For instance, spheroids cultured at low oxygen levels (3%) showed reduced size and increased necrotic areas, while FBS concentrations above 10% promoted more compact spheroids with distinct proliferative and necrotic zones [141]. These findings underscore the sensitivity of 3D models to environmental conditions.

Although in vitro culture systems inherently lack the full physiological context of living organisms, generating three-dimensional tissue-like structures in suspension poses additional challenges. Organoids remain limited in size—typically in the micrometer to low millimeter range—and differ functionally from native tissues, primarily due to the absence of a circulatory system. As their diameter increases (up to ~5 mm after months of culture), nutrient diffusion and waste removal become inefficient, leading to compromised viability of cells in the core [142]. In native tissues, vascular networks not only deliver oxygen and nutrients but also regulate organ function, homeostasis, and regeneration. Similarly, vascularization is critical in organoid cultures to support survival and functionality by facilitating exchange processes [143]. To address these limitations, microfluidic technologies are being integrated into 3D culture systems. These platforms enhance the physiological relevance of organoids by improving nutrient delivery, guiding growth, enabling real-time sensing, and supporting complex co-culture setups. Notably, self-assembling microfluidic devices allow antitumor testing to be conducted directly on the growth platform post spheroid formation, potentially reducing time and cost in optimizing drug regimens during preclinical evaluation. Advancements in 3D bioprinting and microfluidic technologies are also being investigated to develop tumor models that more accurately reflect the tumor microenvironment [144]. 3D bioprinting enables the precise placement of multiple cell types and ECM components, facilitating the formation of complex tissue-like structures that better reflect native tumor architecture. In parallel, microfluidic devices, often referred to as “tumor-on-a-chip” models, can simulate dynamic physiological conditions, such as fluid flow and mechanical forces, closely simulating the in vivo conditions [145,146,147,148]. These advancements enhance the physiological relevance of tumor models, offering greater insights into cancer biology and improving the predictive value of preclinical studies. Tumor-on-a-chip is a powerful device to investigate the EV-mediated crosstalk between tumor spheroids and surrounding healthy tissues, enabling the study of cancer progression, including cell invasion, angiogenesis, and metastasis [149,150].

By embedding them in extracellular matrix-like materials, it is possible to analyze how cancer cells invade surrounding tissues and, therefore, evaluate anti-metastatic agents [151,152]. This setup provides valuable insights into the molecular mechanisms driving cancer spread and opportunities for metastasis-involved pathway studies and, consequently, therapeutic intervention. Moreover, the 3D structure enriches cancer stem cell populations, facilitating studies on tumor initiation, progression, and recurrence, as well as therapies targeting these cells specifically [153,154,155].

Furthermore, tumor-on-a-chip systems are associated with a microfluidic system, making them a perfect platform for drug distribution and effectiveness in an environment that closely mimics in vivo conditions, providing reliable data for preclinical studies [156,157,158,159].

A key advantage of 3D models is their ability to predict the efficiency of anticancer drugs more accurately, as their 3D structure restricts drug penetration, simulating the barrier present in solid tumors involved in drug and immunotherapy resistance. Furthermore, they serve as an important platform for anticancer drug screening, enabling the evaluation of both the cytotoxicity and efficacy of novel therapeutic agents [124,160,161,162]. High-throughput screening methodologies further enhance their utility, allowing for the testing of large libraries of compounds on spheroids, and identifying potential anticancer drugs that might be overlooked using traditional monolayer assays [158,163,164,165].

## 6. EVs from Tumor Spheroids

**Figure 4:**
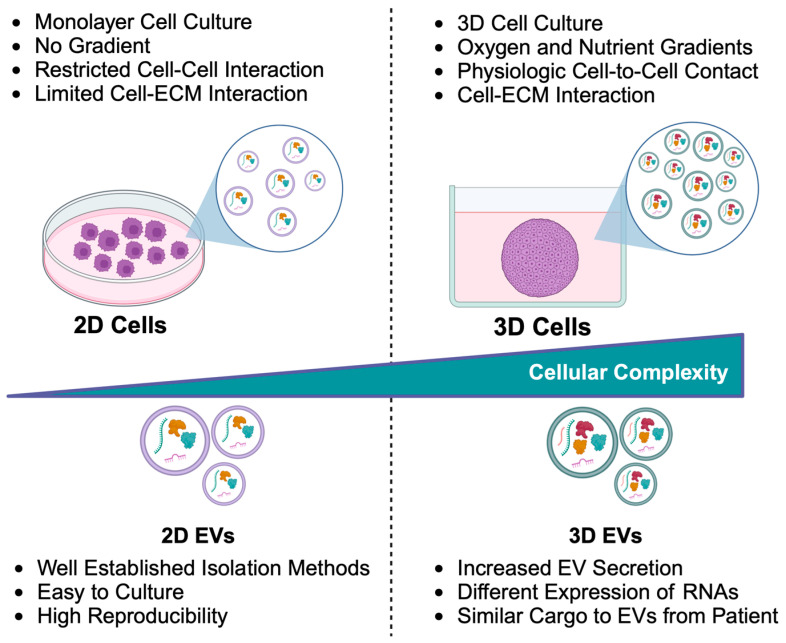
Comparison of 2D and 3D cell culture systems: morphological features and extracellular vesicle (EV) differences. Key distinctions between 2D and 3D culture models in terms of cell morphology, nutrient and oxygen gradients, and the extent of cell–cell and cell–extracellular matrix (ECM) interactions. It also highlights the relative advantages of each system for EV production.

Monolayer cultures have traditionally been widely used for isolating EVs (2D EVs) due to reproducible and well-established isolation methods as well as ease of manipulation. However, limitations of 2D systems have prompted a transition to 3D cultures, such as spheroids, which more accurately reflect in vivo conditions (Figure 4).

It is well documented that 3D cultures not only increase EV secretion but also alter EV composition, particularly by enriching their RNA cargo [166,167,168]. For example, mesenchymal stem cell (MSC) spheroids show increased EV production and altered RNA content compared to their 2D culture counterparts [169]. The cytokine secretion profile of MSC spheroids is influenced by their size: larger spheroids tend to release more pro-angiogenic and anti-inflammatory factors, while smaller spheroids mitigate cellular senescence, promoting the release of pro-angiogenic molecules. Additionally, 3D cultures also enhance the ability of MSCs to selectively release signaling factors through EVs, bolstering their immunomodulatory effects [170]. Kim and colleagues compared the cargo composition of EVs produced from 2D or 3D MSC culture, performing a comparative molecular profiling using proteomics and microRNA sequencing [171]. In this manner, they identified 224 miRNAs expressed in both systems, but 44 miRNAs expressed only in 2D EVs and 130 exclusive to 3D EVs. Among them, the immunomodulatory cytokine TGF-β1 and let-7b-5p miRNA, which negatively regulate the TLR4/NF-κB pathway, are amply upregulated in 3D systems. In gastric cancer, comparison between 2D and 3D systems showed upregulation of microRNA and downregulation of proteins involved in the ADP-ribosylation factor 6 (ARF6) signaling pathways in EVs produced by spheroids, highlighting the impact of cellular organization on EV biogenesis and molecular content [172].

This enhanced EV functionality has notable therapeutic implications. For instance, EVs derived from human dermal fibroblast spheroids exhibit anti-skin-aging properties on skin, suggesting their potential application in preventing and treating cutaneous aging [173]. Similarly, EVs from dermal papilla cell spheroids, essential for hair growth, are enriched in miR-218-5p, an miRNA that upregulates β-catenin signaling, thereby promoting hair follicle development [174]. In regenerative medicine, secretomes and EVs derived from lung spheroids and MSC spheroids have been investigated for their potential in treating lung injury and fibrosis, with lung spheroid-derived factors showing notable, promising repair capabilities [175].

In cancer research, EVs derived from tumor spheroids (3D EVs) are considered more physiologically relevant than those from 2D cultures (2D EVs). For example, a study on cervical cancer spheroids revealed that 3D EVs exhibit a small RNA profile remarkably similar (~96%) to that of in vivo circulating EVs found in the plasma of cervical cancer patients [176]. This high degree of similarity indicates a more accurate model for investigating tumor biomarkers, drug screening, and understanding the molecular mechanisms of tumor progression and metastasis.

3D cultures have shed light on the role of the tumor microenvironment in EV-mediated intercellular communication. For example, in ovarian cancer spheroids, exposure to cisplatin alters the miRNA cargo of secreted EVs, increasing the migration of bone marrow MSCs (BM-MSCs). These activated BM-MSCs subsequently increase secretion of interleukin-6 (IL-6), interleukin-8 (IL-8), and vascular endothelial growth factor A (VEGFA); promote angiogenesis in endothelial cells; and stimulate the migration of low-invasive ovarian cancer cells. Therefore, cisplatin can facilitate pro-tumorigenic behavior by modulating EV content [177,178]. In addition to secreted EVs, a subset of vesicles is trapped within the multicellular ovarian cancer spheroids known as “inner” EVs, participating in vasculogenic mimicry, a process in which cancer cells form vascular-like, tube-shaped structures. This process allows cancer cells to catch oxygen and nutrients and become independent from the presence of endothelial cells and traditional angiogenetic mechanisms [178]. Moreover, the role of EVs in pancreatic tumor-induced cachexia was investigated by targeting ZIP4, a zinc transporter implicated in tumor growth and metastasis. Silencing ZIP4 leads to reduced expression of the small GTPase RAB27B, which in turn decreases the release of heat shock proteins Hsp70 and Hsp90, two proteins commonly found in EVs [179].

3D cell culture systems have also been employed to investigate the role of the tumor microenvironment and its potential contribution to cancer EV secretion. A study using heterotypic melanoma cells and preadipocytes revealed an exosomal crosstalk between the two cell types that promotes cancer progression and metastasis through miR-155 expression [180]. Such findings may provide insights into using miRNA modulation as a therapeutic strategy to inhibit or treat melanoma. Similarly, EVs released from non-small-cell lung cancer (NSCLC) actively modulate the tumor microenvironment, supporting cancer proliferation and enhancing signaling to inhibit apoptosis, thereby promoting cancer metastasis [181]. Donzelli and coworkers also developed a 3D spheroid heterotypic culture model of NSCLC cell lines and fibroblasts to investigate the interplay between EVs, miR-574-5p, and the inflammatory mediator prostaglandin E2 (PGE2). Therefore, EVs containing miR-574-5p are taken up by neighboring cancer cells, triggering the upregulation of PGE2 synthesis [182]. This highlights the intricate nature of EV interactions and the advantages of using multicellular 3D culture models to clarify mechanisms arising from these complex interactions, potentially targeted for future therapeutic purposes [183]. Further evidence of the microenvironment’s impact on EV secretion has also been observed from the comparison between spheroids and xenograft models. In vitro, multicellular spheroids from patient-derived colorectal cancer secrete higher quantities of EVs, with release occurring from apical and basolateral cancer cell domains. In contrast, xenograft tumor spheroid models demonstrate EV release from all cancer cell domains, and the overall quantity of EVs is significantly greater compared to those released from spheroid cultures [184].

On the other hand, 3D systems also permit the investigation of the role of immune cells in cancer progression, because the interplay between cancer and immune cells via EVs within the TME plays a pivotal role in tumor development and modulation of immune responses [185]. Notably, differences in the composition and function of tumor-derived EV between 2D and 3D cell culture models can significantly impact the anti-cancer immune response. For instance, compared to traditional 2D glioblastoma cultures, extracellular vesicles released by 3D tumor organoids displayed a higher abundance of miRNAs involved in immunoregulatory signaling, such as interleukin-4 (IL-4) and interleukin-13 (IL-13) cytokine pathways. They are involved in tumor proliferation and in immunosuppressive phenotypes by inducing macrophages to polarize into an M2-like phenotype [186]. A difference in EV composition and effect between 2D and 3D systems was also observed in the breast cancer model. EVs from spheroids carried a higher number of pro-inflammatory and pro-tumorigenic molecules related to Natural Killer (NK) cell activation. This was evident when EVs derived from breast cancer spheroids were used to treat peripheral blood mononuclear cells (PBMCs) from healthy donors. This treatment led to both activation and reduction in the proportion of CD335+/CD11b+ Natural Killer (NK) cells, along with a significant decrease in CD39+ regulatory T cells (T-reg) involved in suppressing excessive immune responses related to the inflammatory response [187]. In addition, β-catenin-mutated tumors, which are known to exhibit resistance to immunotherapy, have been further investigated in the context of liver cancer. A recent study analyzing β-catenin activation in liver cancer cell lines and hepatocellular carcinoma patient samples revealed that activation of this pathway correlates with a reduction in exosome release and diminished immune cell infiltration [188].

Spheroids have proven to be invaluable tools for studying cancer stem cells (CSCs) and their role within tumor tissues. In a comprehensive study involving 67 cancer cell lines cultured under 3D conditions, spheroids were classified based on cellular aggregates formed and analyzed for gene expression profiles, with a focus on oncogenes and stem cell genes. Cells forming 3D structures were shown to efficiently secrete tumor EVs positive for epithelial cell adhesion molecule (EpCAM) and HSP90. These EVs were capable of transforming induced pluripotent cells into CSC-like cells, highlighting the functional role in maintaining CSC populations and driving tumor progression [189].

Additionally, cancer-derived EVs contain circular RNA (circRNAs) with significant regulatory roles [190]. A study using a breast cancer spheroid model demonstrated that EVs from cancer cells contain elevated levels of circRNAs compared to EVs from normal breast cells. Among this, a specific circRNA was found to regulate glycolysis in breast cancer cells via miR-1252-5p-mediated regulation of PFKFB2 (6-Phosphofructo-2-Kinase/Fructose-2,6-Biphosphatase 2) expression, emphasizing the metabolic reprogramming potential of EVs from cancer cells [191].

Moreover, 3D culture systems have significantly advanced the study of EVs as potential cancer biomarkers for diagnosis, prognosis, and treatment monitoring [192,193]. Compared to nonneoplastic individuals, cancer patients exhibit a significantly higher concentration of EVs in body fluids, along with altered molecular composition. As a result, the miRNA and protein cargo of EVs can be investigated as promising biomarkers. Since 3D models more accurately simulate the in vivo tumor environment, EVs secreted from 3D cultures closely reflect the molecular signals observed in clinical settings. For instance, EVs derived from gastric cancer spheroids exhibit a general upregulation of microRNAs and a downregulation of protein content, indicating a shift in their molecular profile. These compositional changes influence both the biological activity of EVs and their uptake by recipient cells, making 3D cultures a more physiologically relevant and high-throughput alternative to traditional 2D in vitro systems [172]. Additionally, EVs released by pancreatic ductal adenocarcinoma (PDAC) tumor organoids have been compared with plasma EVs from patients with PDAC, benign gastrointestinal diseases, and chronic pancreatitis. This comparative analysis led to the identification of four EV proteins as potential novel biomarkers for PDAC, demonstrating the clinical utility of 3D culture-derived EVs in cancer diagnosis and prognosis [194].

Despite growing interest, significant challenges remain in translating tumor spheroid-derived extracellular vesicles (3D EVs) into clinically effective therapeutics. While several studies have reported good manufacturing practice (GMP)-compliant and scalable methods for producing 3D EVs, most of these rely on conventional 2D cultures, which limit scalability and physiological relevance [195]. In contrast, 3D micro-patterned well systems offer a promising alternative for large-scale EV production, with evidence showing improved therapeutic outcomes—such as enhanced morphology and connectivity—in preclinical stroke models. To address these limitations, Son and colleagues developed a bioprocessing platform based on a non-adhesive, microwell-patterned 3D culture system optimized for the serum-free production of EVs from human Wharton’s Jelly-derived MSCs (WJ-MSCs) and compared the yield, size distribution, and purity of EVs produced in this 3D system (3D EVs) to those derived from standard 2D MSC cultures (natural EVs), highlighting the advantages of the 3D approach for consistent and scalable EV manufacturing [196].

## 7. EVs from Tumor Organoids

Organoid-derived EVs present unique challenges and complexities not typically encountered with EVs from traditional 2D cell cultures. One major factor is the structural and cellular heterogeneity of organoids, which are composed of multiple cell types. This diversity leads to a wider array of EV subpopulations, each potentially varying in size, morphology, and biological function. Consequently, standard characterization tools like Transmission Electron Microscopy (TEM) must be interpreted with caution due to this added complexity. In addition to their biological intricacy, organoids require advanced culturing systems, usually involving three-dimensional matrices, that further complicate EV isolation. Components of these matrices, including residual proteins or gel fragments, can contaminate EV preparations and hinder downstream analysis. Isolating EVs from organoid systems demands highly refined techniques and greater precision than those used for standard 2D cell culture models to preserve vesicle purity and functionality. Nevertheless, EV studies using organoids have distinct benefits, one of which is the limitation of in vivo experiments using a model that closely resembles the native tissue, with a closer physiologic relevance over 2D culture.

The study of EVs from 3D in vitro culture systems mainly focuses on 3D spheroid tumor models, which have some characteristics that better represent the system, such as the necrotic core. However, organoid-derived EVs are emerging as critical mediators of cancer progression, intercellular signaling, and diagnostic potential, with studies across tumor types offering unique mechanistic insights (Table 1). In pancreatic cancer, particularly pancreatic ductal adenocarcinoma (PDAC), several studies emphasize EVs’ diagnostic and pathogenic relevance. A 2021 study showed that PDAC organoids recapitulate tumor heterogeneity and share EV miRNA signatures with matched patient plasma, including miR-21 and miR-195, while also revealing that extracellular matrix remodeling (e.g., collagen I deposition) boosts EV release in both PDAC and chronic pancreatitis, explaining elevated circulating EV levels in both conditions [197]. Moreover, the implementation, using a 3D biomimetic PDAC model that integrates tumor organoids and host-matching stromal cancer-associated fibroblasts (CAFs), showed that the matrix stiffness activates CAFs to increase exosome secretion, driving chemoresistance, a process reversible by exosome inhibitors like climbazole and imipramine [198]. PDAC with cachexia-related muscle wasting was investigated in 2025 through EVs as a mediator in PDAC–skeletal muscle communication. PDAC-derived EVs enriched in miR-223-5p promote muscle wasting through suppression of METTL14 via MAFA targeting, linking circulating EV miRNA content to cachexia and poor prognosis [199]. Researchers have also analyzed the protein profiles of EVs from PDAC organoids using mass spectrometry to distinguish EVs from PDAC and healthy pancreatic organoids. They observed that tumor-derived EVs are enriched in pro-tumorigenic proteins like LAMA5 and SDCBP, highlighting their utility for early diagnosis and monitoring [200]. In colorectal cancer (CRC), organoid-based studies consistently demonstrate the role of EVs in disease progression and biomarker development. An investigation focusing on the adenoma-to-carcinoma transition highlighted miR-1246 upregulation in CRC organoid-derived EVs, showing its role in promoting proliferation [201]. Another study identified APC, a common tumor suppressor gene, and its mutation is associated with familial adenomatous polyposis (FAP) and sporadic colorectal tumors. APC mutation and collagen deposition are critical enhancers of EV release in CRC organoids, with fibroblast-derived EVs shown to induce organoid colony formation under hypoxia, supporting a reciprocal tumor–stroma EV axis [202]. The researchers knocked out MMP3 in tumor organoids, highlighting MMP3’s dual role in tumoroid structure and EV integrity, showing that MMP3-rich EVs rescue proliferation and CD9/CD63 expression in deficient organoids, thereby positioning MMP3 as a key modulator of tumor EV function [203]. A 2025 multicenter study identified four exosomal miRNAs (miR-4284, miR-5100, miR-1246, miR-1290) elevated in CRC patient serum, showing diagnostic performance comparable to carcinoembryonic antigen (CEA) and improved accuracy when combined [204]. Earlier foundational work in 2013 on LIM1863 colon carcinoma organoids isolated two distinct EV subpopulations (apical and basolateral), with distinct proteomic profiles, including tumor-promoting complexes such as EpCAM, claudin-7, and CD44, demonstrating spatial and functional heterogeneity of EVs [205]. In glioblastoma, a 2024 study comparing 2D and 3D models found that organoid-derived EVs are enriched in immune-modulatory miRNAs and proteins, underscoring 3D models’ advantage in capturing glioblastoma EV biology [186]. Furthermore, studies across multiple cancer types deepen our understanding of EV regulation. An investigation across lung and pancreatic organoids revealed that Wnt signaling is tightly coupled to cell proliferation and EV secretion, with this link disrupted in PDAC but preserved in lung adenocarcinoma, emphasizing tissue-specific EV regulatory mechanisms [206].

Collectively, these studies demonstrate that organoid-derived EVs provide a robust platform for elucidating tumor biology, intercellular signaling, and biomarker discovery, with their context-specific cargo and behavior offering powerful avenues for precision oncology.

## 8. Conclusions

The transition from 2D to 3D cell culture systems represents a significant advancement in EV research, providing more physiologically relevant models that closely mimic the complexities of in vivo environments. While 2D monolayer cultures have been instrumental due to their reproducibility and ease of EV isolation, they fail to capture the intricate cellular interactions and microenvironmental dynamics of native tissues. In contrast, 3D cultures, such as spheroids and organoids, overcome these limitations, offering a closer mimicry of native tissue structures and enhancing the biological relevance of EV studies. Moreover, 3D culture techniques enable the growth of larger cell quantities in space- and cost-efficient ways, which are essential for the clinical application of EV-based therapies.

Spheroids derived from cancer cells showed increased EV production, with altered RNA content and cytokine profiles depending on spheroid size and composition. These characteristics have broadened the therapeutic potential of EVs for applications in tissue repair, immune modulation, and targeted therapy, as evidenced by their roles in lung injury and skin anti-aging studies. Notably, 3D-derived EVs from tumor spheroids more closely resemble those found in cancer patients, making them invaluable for studying tumor progression, metastasis, and therapeutic responses.

Organoid-derived EVs significantly deepen the landscape of EV research by more accurately recapitulating tissue-specific architecture and the complex multicellular interactions present in native tumors. This enables more precise investigation of tumor–stroma crosstalk, immune modulation, and mechanisms of drug resistance. These systems have enabled the discovery of cancer-specific EV cargo with strong potential as diagnostic and prognostic biomarkers. However, their biological complexity also introduces technical challenges in EV isolation and characterization, particularly due to matrix contamination and heterogeneity of vesicle populations.

In conclusion, EVs released by 3D tumor models, including spheroids and organoids, represent a promising area of study with significant implications for understanding tumor biology and developing novel cancer therapies. These vesicles not only provide a window into the complex intercellular communication within tumors but also offer potential as biomarkers and therapeutic targets. Future efforts should address the challenges in EV isolation, large-scale production, and characterization to facilitate their translation into clinical applications. By bridging the gap between in vitro and in vivo systems, 3D cultures enable more accurate modeling of disease processes and offer a powerful platform for developing targeted, effective cancer treatments.

## Figures and Tables

**Table 1:** EVs from tumor 3D spheroids and organoids.

Tumor	Cancer Cells	3D Model	3D EV Isolation Method	Finding	References
Gastric cancer	MKN74 MKN45	Spheroid	Differential ultracentrifugation	Upregulation of microRNAs and downregulation of proteins in 3D EVs	[172]
Cervical cancer	Hela	Spheroid	Filtration	EV small RNAs	[176]
Ovarian cancer	HeyA8 Ovcar3	Spheroid	Differential ultracentrifugation	Pro-angiogenetic role	[177]
Ovarian cancer	CABA I	Spheroid	Differential ultracentrifugation	“Inner” EVs	[178]
Pancreatic cancer	AsPC-1 BxPC-3	Spheroid	Isolation kit	ZIP4 knockdown reduced EVs’ pancreatic cancer release	[179]
Melanoma	B16	Spheroid	N/A	Influence of preadipocytes in melanoma growth	[180]
Lung cancer	3LL A549	Spheroid	Differential ultracentrifugation	Modulation of tumor microenvironment	[181]
Lung cancer	A549 H1650 2106T	Spheroid	Differential ultracentrifugation	Role of miR-574-5p in prostaglandin H_2_ regulation	[182]
Colorectal cancer	Primary	Spheroid	N/A	Multilayer spheroids release more EVs	[184]
Glioblastoma	IDH wild-type (CNS WHO grade 4)-derived models (BTIC10, -13, -131, -18, -129, -155)	Organoid	Precipitation and immunoaffinity	Comparison between EVs released from 2D and 3D models	[186]
Breast cancer	HS578T BT474	Spheroid	Differential ultracentrifugation	Effects of EVs breast cancer on PBMC from healthy donors	[187]
Hepatocellular carcinoma	HepG2 Huh7	Spheroid	Differential ultracentrifugation	β-catenin decreases EV release and immune cell infiltration	[188]
Prostatic adenocarcinoma	PC-3	Spheroid	Differential ultracentrifugation Filtration	Secretion of HSP90 and EpCAM	[189]
Breast cancer	MDA-MB-231	Spheroid	Differential ultracentrifugation	circCARM1 promotes breast cancer proliferation and glycolysis	[191]
Pancreatic ductal adenocarcinoma	Primary cells	Spheroid	Filtration and ultracentrifugation	New biomarkers	[194]
Pancreatic ductal adenocarcinoma	PDAC cell lines (derived from primary tumors)	Organoid	Differential ultracentrifugation	miRNA EVs released with matched patient plasma and extracellular matrix remodeling	[197]
Pancreatic ductal adenocarcinoma	Mouse-derived organoids	Organoid	Isolation kit	3D biomimetic PDAC model with integrated CAF	[198]
Pancreatic ductal adenocarcinoma	Patient-derived organoids	Organoid	Differential ultracentrifugation	Absorption of miRNA in PDAC-derived EVs by skeletal muscles and the role in cachexia	[199]
Pancreatic ductal adenocarcinoma	Patient-derived organoids	Organoid	Isolation kit	Differences between EVs from PDAC organoids and healthy pancreatic organoids	[200]
Colorectal cancer	Patient-derived organoids	Organoid	Differential ultracentrifugation	The role of miR-1246 in promoting proliferation	[201]
Colorectal cancer	Mouse- and patient-derived organoids	Organoid	Isolation kit	APC mutation and collagen deposition enhance EV release	[202]
Colorectal cancer	LuM1 cell line	Organoid	Differential centrifugation and concentration	MMP3 knockout led to the additional release of EVs from organoids	[203]
Colorectal cancer	Patient-derived organoids	Organoid	Differential ultracentrifugation and filtration	miR-4284, miR-5100, miR-1246, miR-1290 elevated	[204]
Colorectal cancer	Human colon carcinoma LIM1863 cells	Organoid	Isolation kit	EVs isolated from apical and basolateral region have distinct proteomic profiles	[205]
Pancreatic ductal adenocarcinoma Lung bronchiolar Lung adenocarcinoma	Human PDAC organoids Mouse pancreas ductal and lung organoids Human bronchiolar and LUAD organoids	Organoid	Differential ultracentrifugation	Wnt signaling is tightly coupled to cell proliferation and EV secretion in lung adenocarcinoma but disrupted in PDAC	[206]

## Abbreviations

The following abbreviations are used in this manuscript:

2D	Two-dimensional
3D	Three-dimensional
3D EVs	Tumor spheroid-derived extracellular vesicles
ARF6	ADP-ribosylation factor 6
ASCs	Adult tissue-resident stem cells
BMDC	Bone marrow-derived cells
CAF	Cancer-associated fibroblast
CEA	Carcinoembryonic antigen
CRC	Colorectal cancer
ECs	Endothelial cells
ESCRT	Endosomal Sorting Complex Required for Transport
ESCs	Embryonic stem cells
EVs	Extracellular vesicles
FAP	Familial adenomatous polyposis
FBS	Fetal bovine serum
Fc	Fragment crystallizable
FLT1	Vascular endothelial growth factor receptor
GBM	Glioblastoma
GelMA	Gelatin methacrylate
GMP	Good manufacturing practice
HA	Hyaluronic acid
HSC	Hepatic stellate cell
IL-4	Interleukin-4
IL-6	Interleukin-6
IL-8	Interleukin-8
IL-13	Interleukin-13
ILV	Intraluminal vesicle
iPSCs	Induced pluripotent stem cells
MDSC	Myeloid-derived suppressor cell
METTL3	Methyltransferase-like 3
MIO NPs	Magnetic iron oxide nanoparticles
MMPs	Matrix metalloproteinases
MTS	Multicellular tumor spheroids
MVBs	Multivesicular bodies
MVs	Microvesicles
MSCs	Mesenchymal stromal cells
NGF	Nerve growth factor
NGFR	NGF receptor
NK	Natural Killer
PBMCs	Peripheral blood mononuclear cells
PD-1	Death protein 1
PDAC	Pancreatic ductal adenocarcinoma
PD-L1	Programmed death-ligand 1
PDOs	Patient-derived organoids
pHEMA	Poly(2-hydroxyethyl methacrylate)
PMN	Pre-metastatic niches
SNARE	SNAP receptor
SNAP	Soluble NSF [N-ethylmaleimide-Sensitive Factor] attachment protein
TAM	Tumor-associated macrophages
TEM	Transmission Electron Microscopy
TME	Tumor microenvironment
TMZ	Temozolomide
TNBC	Triple-negative breast cancer
T-reg	Regulatory T cells
VEGF	Vascular endothelial growth factor
WJ-MSCs	Wharton’s Jelly-derived MSCs
